# TOWARD A GLOBAL DEFINITION OF PERSON-CENTERED DEMENTIA CARE

**DOI:** 10.1093/geroni/igae098.0679

**Published:** 2024-12-31

**Authors:** Sam Fazio, Walter Moczygemba, Lauren Stratton, Sheryl Zimmerman

**Affiliations:** Alzheimer’s Association, Chicago, Illinois, United States; University of North Carolina, Chapel Hill, North Carolina, United States; Alzheimer’s Association, Chicago, Illinois, United States; University of North Carolina, Chapel Hill, North Carolina, United States

## Abstract

Person-centeredness is instrumental to quality dementia care, yet there remains inconsistency surrounding its definition and attempts to unify disparate conceptualizations are often based on high-income and Eurocentric perspectives. To address this, we surveyed 39 Alzheimer’s Disease International members on their perspectives and experiences with quality of dementia care, definitions of person-centered dementia care, and the challenges and successes of person-centered dementia care in their countries. Overall, respondents describe a lack of global quality dementia care, naming several barriers to its successful implementation. Respondents also agreed the creation of a universal definition of person-centered dementia care is necessary for furthering high quality dementia care globally. Five core tenets representative of the geographically and economically diverse sample were identified to be included in such a definition: individualize care, value the individual, empower the individual, acknowledge the importance of caregivers, and address dimensions of wellness. Discussion will focus on recommendations for practitioners, researchers, and policy advocates on how to embody global tenets of person-centered dementia care. Additionally, internationally common barriers to person-centered dementia care are identified for further study and advocacy efforts.

